# Evaluation of Suzor forceps training by studying obstetric anal sphincter injuries: a retrospective study

**DOI:** 10.1186/s12884-020-03358-0

**Published:** 2020-11-10

**Authors:** Perrine COSTE MAZEAU, Nedjma BOUKEFFA, Nathalie TICAUD BOILEAU, Samantha HUET, Maud TRAVERSE, Jean-Luc EYRAUD, Alexine LAGUERRE, Cyrille CATALAN, Cécilia RIEDL

**Affiliations:** 1 Department of Gynecology and Obstetrics, Mother and Child Hospital, Limoges Regional University Hospital, 8 avenue Dominique Larrey, 87000 Limoges, France; 2 Department of Gynecology and Obstetrics, Mont-de-Marsan Hospital Center, 417 Avenue Pierre de Coubertin, 40024 Mont-de-Marsan, France

**Keywords:** Instrumental delivery, Obstetric anal sphincter injuries, Residents, Suzor forceps

## Abstract

**Background:**

Instrumental deliveries are an unavoidable part of obstetric practice. Dedicated training is needed for each instrument. To identify when a trainee resident can be entrusted with instrumental deliveries by Suzor forceps by studying obstetric anal sphincter injuries.

**Methods:**

A French retrospective observational study of obstetric anal sphincter injuries due to Suzor forceps deliveries performed by trainee residents was conducted from November 2008 to November 2016 at Limoges University Hospital. Perineal lesion risk factors were studied. Sequential use of a vacuum extractor and then forceps was also analyzed.

**Results:**

Twenty-one residents performed 1530 instrumental deliveries, which included 1164 (76.1%) using forceps and 89 (5.8%) with sequential use of a vacuum extractor and then forceps. Third and fourth degree perineal tears were diagnosed in 82 patients (6.5%). Residents caused fewer obstetric anal sphincter injuries after 23.82 (+/− 0.8) deliveries by forceps (*p* = 0.0041), or after 2.36 (+/− 0.7) semesters of obstetrical experience (*p* = 0.0007). No obese patient (body mass index> 30) presented obstetric anal sphincter injuries (*p* = 0.0013). There were significantly fewer obstetric anal sphincter injuries after performance of episiotomy (*p* <  0.0001), and more lesions in the case of the occipito-sacral position (*p* = 0.028). Analysis of sequential instrumentation did not find any additional associated risk.

**Conclusion:**

Training in the use of Suzor forceps requires extended mentoring in order to reduce obstetric anal sphincter injuries. A stable level of competence was found after the execution of at least 24 forceps deliveries or after 3 semesters (18 months) of obstetrical experience.

## Background

Instrumental deliveries are an unavoidable part of obstetric practice, with an operative delivery rate that depends on the medical center (varying from 5.3 to 34.1% of all deliveries) [1]. The choice of the instrument differs depending on local habits and personal selection. The key instrument at the Limoges University Hospital is the Suzor forceps (SF; forceps with parallel shafts). The second choice is the Kiwi OmniCup vacuum extractor (VE) and Thierry’s spatula is hardly used.

The main maternal complications associated with forceps delivery (FD) are perineal tears, which are more frequent than with the VE [2, 3]. Perineal tears can be classified into four categories of severity [4]. In the long run, anal incontinence appears to be more common after FD than after VE or spontaneous vaginal delivery [5–7].

The main purpose of the study was to determine how many operative deliveries by Suzor forceps, and how many semesters (or months), it would take for a resident to be entrusted with instrumental delivery, by evaluating the rate of obstetric anal sphincter injuries (OASIS). Maternal, obstetrical and fetal factors that could affect the outcome of perineal complications were also studied.

## Methods

This retrospective study was conducted in the Gynecology and Obstetrics Unit of the Limoges Mother and Child Hospital (LMCH), in France, a level 3 maternity unit, from November 1st 2008 to November 1st 2016. The study was approved by the Limoges Regional University Hospital institutional review board (306–2019-72).

The authors retrieved each resident’s teaching plan, including every semester completed until then. Residents were assumed to be the main delivering physicians every time their names were written on the patient’s file. The authors considered the first semester of attendance at LMCH to be the resident’s first level of experience. Each resident increased his/her obstetrical experience from one level to another every semester during which he/she performed FD at LMCH.

Inclusion criteria were instrumental deliveries using the SF, or sequential use of the Kiwi OmniCup° vacuum and then forceps by residents who started their training after November 2008, without mentoring by a senior physician, in singleton pregnancies with a cephalic presentation, regardless of the term or the level of engagement of the fetus in the genital tract. Sequential use of the vacuum extractor and then forceps was studied as well (VESF).

FD that involved a senior physician were excluded, as were failed operative deliveries, twin pregnancies, fetuses in the breech or transverse position, in utero fetal death, and medical termination of pregnancy. All files with incorrectly filled reports or that neglected to state clearly whether the senior physician participated in the instrumental delivery were also excluded.

The main outcome was the rate of severe perineal tears, 3rd or 4th degree, which are considered as obstetric anal sphincter injuries (OASIS). Sultan’s classification, used since 2007 by the Royal College of Obstetricians and Gynaecologists [4], was used to grade the perineal lesions. These OASIS were clinically suspected by residents and confirmed by a senior obstetrician.

Several risk factors for OASIS were studied (delivering physician’s experience, and maternal, fetal and obstetrical criteria). Obstetric practices did not change during the study period (episiotomy policy, for example).

Data were analyzed using JMP 12.0.1 software (SAS Institute, Cary, USA) and the results were presented as mean values, medians, standard deviations and percentages. Univariate and multivariate analysis was performed to assess the risk of OASIS given the obstetric experience of the delivering physician, controlling for confounding factors. Comparison of variables was done using the Chi-2 test with a significant cut-off determined as 5%. Odds ratios were implemented for some variables. A logistic regression analysis was performed.

## Results

**Fig. 1:**
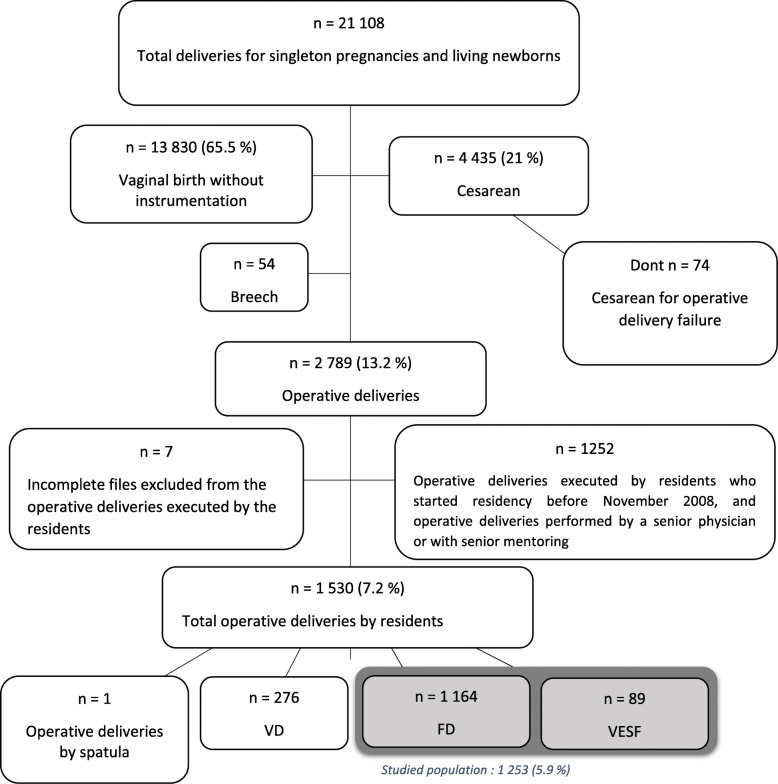
Deliveries at Limoges Mother and Child Hospital from November 1st 2008 to November 1st 2016

During the 8-year study period, 21 residents performed 1253 instrumental deliveries, which represented 45% of all instrumental deliveries. Forceps were chosen as the delivery instrument in 1164 cases (92.9%), and in 89 cases (7.1%) the VE was used first and then forceps (Fig. 1).

At the time of the study, 10 senior residents completed their training and 11 junior residents were still undergoing training. Each of them had attended the LMCH for at least one semester during which they performed at least one FD.

**Fig. 2:**
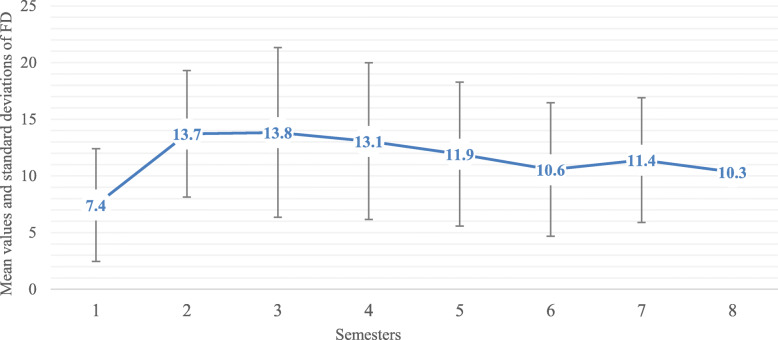
Mean values and standard deviations of forceps delivery according to the experience of the resident

One resident performed on average 11.6 (+/− 6.5) FD each semester. Older residents performed more instrumental deliveries per semester than younger ones (840 FD in total for the older residents and 12.9 FD per semester (± 6.4) vs 413 and 9.6 (± 6.0) per semester for the younger residents)*.* The number of instrumental deliveries performed by residents each semester increased with their seniority and thus so did their obstetrical and instrumental experience *(*Fig. 2*)*.

The mean time lapse before the first operative delivery by forceps was 84 days (+/− 81 days). The older residents took on average 49 days before their first use of forceps, and younger residents about 116 days (95% CI [53–180]; *p* <  0.018) *(*Table 1*)*.

**Table 1:** Time (in days) before first forceps delivery in the Limoges Mother and Child Hospital

	Mean value	Median	Standard deviation
Older residents	49	39.5	44.5
Younger residents	116	89	95
**All**	84	65	81

### Characteristics of patients and newborns

The characteristics of patients and newborns are described in Table 2.

**Table 2:** Characteristics of patients and newborns

Variables		
Age. *median (standard deviation)*	29.2	(± 5.3)
BMI. *median (standard deviation)*	22.3	(± 4.5)
Primiparity. *n (%)*	995	(79.4%)
Scarred uterus. *n (%)*	136	(10.8%)
Analgesia. *n (%)*	1243	(99.2%)
Indication of instrumental delivery:		
Abnormal fetal heart. *n (%)*	758	(60.5%)
Arrest of fetal descent. *n (%)*	489	(39.0%)
Contraindications to expulsive efforts. *n (%)*	6	(5.8%)
Induced labor. *n (%)*	350	(27.9%)
Progression of the fetus at the time of the first instrument used:		
Pelvic inlet. *n (%)*	158	(12.6%)
Midpelvis. *n (%)*	1022	(81.6%)
Pelvic outlet. *n (%)*	73	(0.06%)
Operative delivery in the occipito-sacral position. *n (%)*	84	(6.7%)
Intact perineum. *n (%)*	10	(0.8%)
Perineal tears:		
1st degree. *n (%)*	80	(6.4%)
2nd degree. *n (%)*	82	(6.5%)
3rd degree. *n (%)*	70	(5.6%)
4th degree. *n (%)*	12	(1.0%)
Episiotomy. *n (%)*	1128	(90.0%)
Episiotomy without any other associated lesion. *n (%)*	999	(79.7%)
Blood loss over 500 mL. *(%)*	74	(6.3%)
Newborn’s weight in grams. *Mean (standard deviation)*	3277	(± 439)
Newborn’s weight ≥ 4000 g. *n (%)*	54	(4.3%)
Cranial perimeter (cm). *median (standard deviation)*	35	(± 1.2)
Gestational age at delivery in weeks of gestation. *Median (standard deviation)*	40	(± 1.4)
Premature births before 37 weeks of gestation. *n (%)*	53	(4.2%)

### Risk factors for perineal tears

The risk factors for OASIS were: delivering physician’s experience, and maternal, fetal and obstetrical criteria (Table 3).

**Table 3:** Identification of risk factors for obstetric anal sphincter injuries (3rd or 4th degree perineal tear)

	*p*
***Resident’s experience:***	
Resident who performed fewer than 24 forceps deliveries	**0.0041**
Resident who completed fewer than 2.36 semesters of experience practicing instrumental deliveries	**0.0007**
***Obstetrical factors:***	
Protective effect of episiotomy	**< 0.0001**
Posterior presentation	**0.028**
Progression of the fetus during the fitting of the first instrument	0.196
Induced labor	0.98
Indication of the operative delivery	0.12
***Maternal factors:***	
Protective effect of obesity (BMI ≥ 30)	**0.0013**
Scarred uterus	0.97
Primiparity	0.18
***Fetal factors:***	
Cranial perimeter < 330 mm	**0.03**
Fetal macrosomia (≥ 4000 g)	0.10
Post-term delivery (≥ 41 weeks of gestation)	0.17

### Perineal lesion rate according to the resident’s experience

**Fig. 3:**
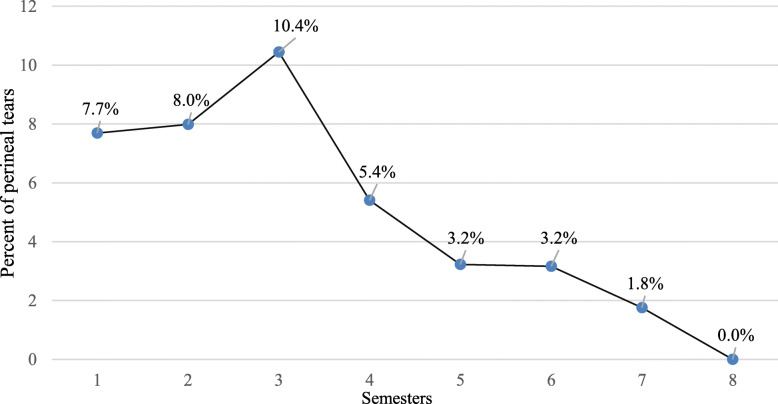
Evolution of the rate of obstetric anal sphincter injuries according to the experience of the resident

Eighty- two OASIS (6.5%) occurred in the 1253 FD in the immediate postpartum period. There was an overall decrease in the rate of OASIS as the resident’s obstetrical experience increased: 7.7 to 10.4% OASIS were observed from the 1st to the 3rd semester of obstetrical experience, and then 5.4 to 0% OASIS from the 4th to the 8th semester *(*Fig. 3*)*.

Sixty-one (8.8%) OASIS among 693 FD were identified during the 1st to 3rd semester of obstetrical experience versus 21 (3.7%) among 560 FD during the 4th to 8th semester of experience (*p* = 0.0002; OR 2.37; 95% CI [1.42–3.94]). Residents caused statistically fewer OASIS after 2.36 semesters of experience (*p* = 0.0007) and after 23.82 (+/− 0.8) FD according to the logistic regression analysis (*p* = 0.0041).

### Obstetrical risk factors for perineal tears

Episiotomy was performed in 90% of the patients. An obstetric anal sphincter injury occurred in 4.6% of these patients, versus 24% for the patients who did not have mediolateral episiotomy (*p* < 0.0001). There were significantly fewer OASIS in cases of episiotomy (OR 0.15; 95% CI [0.09–0.25]).

The occipito-sacral position during birth was observed in 6.7% of FD. This position was significantly associated with a higher rate of OASIS, with 16.7% versus 5.9% for the occipito-pubic position (*p* = 0.028; OR 2.92; 95% CI [1.54–5.53]).

The most frequent level of instrument application was midpelvis (81.6%). There were more OASIS (11%) concerning FD in the pelvic outlet, with no significant difference between operative deliveries performed in the midpelvis or in the pelvic inlet (*p* = 0.196).

There was no link between OASIS and an indication for operative delivery (abnormal fetal heart rate, lack of pushing or medical contraindication to pushing) (*p* = 0.12), or between induced labor and the rate of OASIS (*p* = 0.98).

Almost all patients underwent epidural anesthesia. No sub-group analysis was performed.

### Maternal risk factors for perineal tears

No obese patient (BMI ≥30) presented OASIS, while overweight patients (BMI between 25 and 30) (7.2%), normal weight patients (BMI between 18.5 and 25) (7.4%) and underweight patients (BMI < 18.5) (5.7%) showed a statistically significant difference (*p* = 0.0013), even after multivariate analysis.

Primiparous patients represented 79.4% of the studied population and did not present significantly more OASIS than multiparous women (*p* = 0.18).

Patients with a previous cesarean section had no higher risk of OASIS (6.5 vs 6.6%; *p* = 0.97).

### Fetal risk factors for perineal tears

There were significantly more OASIS among mothers of newborns with a cranial perimeter under 330 mm (14%) than above 330 mm (6.3%) (*p* = 0.03) (OR 2.21; 95% CI [1.15–5.07]).

There were 12.3% OASIS in the case of fetal macrosomia (≥ 4000 g) versus 6.3% for mothers of newborns under 4000 g in weight, the difference not being significant (*p* = 0.10).

There were more OASIS for post-term labor (> 41 weeks of gestation (WG)) in comparison to newborns at term (37–41 WG) and premature births (< 37 WG), but the difference was not significant (*p* = 0.17). When analyzing birth weight as a confounding factor, the group under 41 WG showed a mean weight of 3198 g (± 427) (95% CI [3171–3226]) versus 3509 g (± 390) (95% CI [3466–3552]) for post-term babies (*p* < 0.0001).

### Maternal morbidity

Blood loss over 500 mL was significantly more frequent in patients with OASIS (*p* = 0.01; 13.5% vs 5.4%) (OR 2.74; 95% CI [1.34–5.61]).

### Subgroup analysis: delivery by vacuum extractor then Suzor forceps (VESF)

A subgroup analysis of perineal lesions in the case of sequential instrumentation compared to operative delivery by forceps alone showed that the rate of OASIS during VESF was 5.6% versus 6.6% for FD alone (95% CI [5.8–7.1]) (*p* = 0.7).

## Discussion

At Limoges university hospital, the operative delivery rate was approximately 13.2% *(*Fig. 1*)*, which is consistent with the rate of 12% reported in national perinatal surveys in France, a rate which has been stable since the 1980s, and with the wide range of values among centers reported in the international literature: 5.3 to 34.1% [1–8]. The key instrument at LMCH is the Suzor forceps, the second choice is the Kiwi Omnicup vacuum extractor, and Thierry’s spatula is hardly used. Although questionable, the choice of this instrument depends on local habits and personal selection. Contraindicated in some countries, the sequential use of VE and then forceps is controversial in France and occasionally practiced in our maternity unit.

Few studies have focused on the learning curve of residents concerning operative delivery. A team in Nice (south of France), reported a cut-off of 20 operative deliveries using Thierry’s spatula after which residents subjectively felt that they could perform operative deliveries without supervision [9]. The same team also showed in another study based on objective criteria, such as those used in the present study (maternal perineum), that there was an additional risk of OASIS if the operative delivery by spatula was performed by an inexperienced resident compared to a resident who had completed at least 5 semesters [10].

In the present study, we found that residents caused statistically fewer OASIS after 2.36 semesters (14 months) or after 24 FD. A clear decrease in OASIS was noted between the 3rd and 4th semesters *(*Fig. 3*).* These results are therefore comparable to those of the team in Nice [9]. In fact, the ranking of the different semesters of attendance by the residents started with the first semester of training at LMCH, which is mostly during the 2nd or 3rd semester of the whole residency program. Given the increasing number of residents in each year, the 4th semester of obstetrical experience in Limoges would match Nice’s 5th semester, which is when the authors of the Nice study consider residents to be sufficiently trained to perform operative deliveries.

The OASIS rate after at least 3 completed semesters was about 3.7%, which matched the rates reported in the literature after FD. A study of 284,783 births in Holland showed stage 3 and 4 perineal tear rates of 1.7% without operative delivery, 4.6% with FD, and 7.8% after VESF [11].

There are few recommendations about the number of operative deliveries to be performed by residents before the empowerment phase. Dupuis et al. consider that 40 operative deliveries including VE, FD, and breech delivery are needed [12]. In our hospital, this would be possible after 3 completed semesters, since a resident performs on average 10 FD per semester (for the new generation), but also operative delivery by VE or breech delivery.

Instrumental delivery is conventionally taught via bedside training, notably with close supervision of the resident by a senior physician. This is referred to as ‘mentoring’. However, because of the significant maternal and fetal morbidity that can be caused by FD, it is essential that it is “never the first time on the patient”. There are many questions concerning ways to improve training of obstetricians without increasing the risks for patients and their newborn. Furthermore, mindset changes concerning the medical field and legal aspects do not facilitate the learning curve [10].

The establishment of a ‘resident log’ of all instrumental deliveries (simulated and in vivo) would allow the senior physician to evaluate the experience of the mentored resident. Furthermore, it would let residents be entrusted with instrumental deliveries by forceps, as long as they had performed at least 24 operative deliveries with close mentoring [13]. There is also the additional optional training of simulated instrumental delivery. In the near future, the French national training program in obstetrics will include obstetrical simulation sessions.

The increasing number of medical students admitted to medical school raises new issues: it is becoming harder during residency to accumulate the minimum number of operative deliveries required for self-sufficiency. The mean number of operative deliveries per semester subsequently decreases with each new generation, as already observed in the present study.

To date, in France, there is no performance evaluation allowing an operator to be declared as fit to perform operative deliveries, or not. Nevertheless, each instrument (vacuum, forceps, spatula) requires proper theoretical and practical training in order to master its specific and proper use (handling, articulation, positioning, and traction).

The practical recommendations of the French national college of obstetricians and gynecologists in 2008 [5] stipulate that teaching and learning of operative delivery must include teaching of the use of forceps, vacuum and spatulas, as these instruments are complementary, and acknowledge that the dangers of operative deliveries are related to the experience of the operator performing them. Knowledge of 2 operative delivery methods is recommended [14, 15], and the choice of instrument should be guided by the clinical indications and not by the operator’s preferences.

The inclusion of simulation in teaching programs would help to raise the number of operative deliveries per resident and would enable experience to be acquired outside an emergency context, while evaluating the performance of the obstetricians during their initial and further education. Vieille et al. proved that it would allow residents to achieve a gain in both theoretical knowledge and practical skills [16]. Dupuis et al. observed the necessity of performing 31 FD in the occipito-posterior position and 62 in the inclined position, at least in simulation, in order to master operative delivery [12]. Improvement of residents’ training programs would eventually lower the rate of avoidable fetal and maternal complications [13]. Access to simulation could make it possible to become empowered more quickly. In the USA, simulation is well established [17]. It is a proper teaching tool and simulation centers work as a network to enhance their resources. It is used to certify health professionals and to accredit medical centers giving proper references. In France and in Europe, those centers lack resources even though they are increasing in prevalence [18]. The national health authority plans to promote the expansion of simulation.

An operator’s lack of experience is not the only cause of fetal and maternal morbidity and mortality. Other known OASIS risk factors were analyzed in our study [10–16, 18–24]. We found statistically more OASIS for FD in the posterior position, in line with the literature data [20, 21]. The systematic use of intrapartum ultrasound to detect fetal position could be helpful in order to decrease OASIS by optimization of the direction of the operative delivery. A protective effect of medio-lateral episiotomy was observed. Concerning this particular factor, studies are inconsistent. Several reviews have shown no positive effect of systematic episiotomy on OASIS [22–25]. Others report that reducing the indication for episiotomy would raise the rate of first and second degree perineal tears, but not third and fourth degree perineal tears [26]. De Leeuw et al. reported a protective effect of episiotomy on the perineal area during FD [27]. Their article was widely discussed in France, because it went against the national clinical recommendations of 2006 concerning instrumental deliveries (against the liberal use of episiotomy during instrumental deliveries; allow the operator to assess clinically whether or not to use episiotomy) [28]. Nowadays in France, whether or not an episiotomy is performed during instrumental delivery still depends on the operator’s clinical assessment during the birth. In our study, there was no episiotomy in 10% of cases, because of the resident’s clinical assessment or a lack of time resulting in limited manual perineal control, which is essential to decrease the risk of OASIS [29].

In our study, more OASIS were reported in patients who gave birth to newborns with a small cranial perimeter. These results are inconsistent with the literature, where increase in cranial perimeter is associated with a statistically higher rate of OASIS [13]. We hypothesize that in such cases there may be dystocia (transverse or improperly flexed position), which complicates the manipulation of forceps, with a different axis of traction, which is riskier for the maternal perineal area.

Obesity (BMI ≥30) was protective against OASIS and was still significant after multivariate analysis (*p* = 0.0013). Nevertheless, obese patients have a higher risk of conceiving macrosomic newborns, a well-known risk factor for OASIS [30]. Two studies on large populations also showed that obesity was protective against OASIS [31, 32]. This could be explained by the fact that obese patients have a larger anus-to-vulva distance because of their fat tissue. Indeed, a short anus-to-vulva distance is a recognized risk factor for OASIS [33].

In our study, there was no significant change in the rate of OASIS according to other risk factors usually associated with OASIS: macrosomia, primiparity, instrumental delivery practiced in the pelvic inlet position. Shoulder dystocia was not studied because of its low incidence (0.5 to 1% of vaginal births) [34]. Maternal ethnic origin is sometimes reported as a factor implicated in maternal perineal complications (Indian and Asian women) [19]. Such factors were not taken into account here, as these ethnicities are not widely represented in our region. Maternal blood loss was also significantly higher in the case of OASIS, which would increase morbidity.

The present study in 1253 cases of FD is the largest to date. However, it has some limitations as it was single-center and retrospective. It was also difficult to study improvement in residents’ FD during semesters spent in other medical centers (4 of them). However, this bias had few effects on the main criteria since residents usually complete those semesters at the end of their training, which means long after the three semesters (18 months) of training for FD in Limoges and after conducting 24 or more FD.

Further analyses could be performed in order to compare perineal outcome after FD in Limoges to another university hospital where simulation is a proper part of the training program, and where residents are assisted by a senior physician until their last semester. Experience with SF should not be generalized to other instruments.

## Conclusion

Operative deliveries by SF can occasionally cause OASIS, which involve a substantial risk of maternal morbidity.

Residents should receive proper practical and theoretical training, including good knowledge of instruments. Our results should encourage obstetricians to update their methods of teaching operative delivery, with proper mentoring and empowerment of residents after completion of at least 24 FD or 3 semesters (18 months) under a senior physician’s supervision.

Simulator training could be a way to raise residents’ experience and, therefore, to lower maternal morbidity in the immediate postpartum period.

## Data Availability

The datasets used and/or analyzed during the current study are available from the corresponding author on reasonable request. All data generated or analyzed during this study are included in this published article.

## Abbreviations

BMI: Body mass index
FD: Forceps delivery
LMCH: Limoges mother and child hospital
OASIS: Obstetric anal sphincter injuries
VESF: Sequential use of the vacuum extractor and then Suzor forceps
SF: Suzor forceps
VD: Vacuum delivery
VE: Vacuum extractor
